# Antioxidant capacity of lipid- and water-soluble antioxidants in dogs with subclinical myxomatous mitral valve degeneration anaesthetised with propofol or sevoflurane

**DOI:** 10.1186/s12917-020-02529-7

**Published:** 2020-08-24

**Authors:** Katerina Tomsič, Alenka Nemec Svete, Ana Nemec, Aleksandra Domanjko Petrič, Tatjana Pirman, Vida Rezar, Tomaž Vovk, Alenka Seliškar

**Affiliations:** 1 Small Animal Clinic, Veterinary Faculty, University of Ljubljana, Gerbičeva 60, 1000 Ljubljana, Slovenia; 2 Department of Animal Science, Biotechnical Faculty, University of Ljubljana, Groblje 3, 1230 Domžale, Slovenia; 3 Faculty of Pharmacy, University of Ljubljana, Aškerčeva 7, 1000 Ljubljana, Slovenia

**Keywords:** Dog, Cardiac disease, Antioxidant capacity, Anaesthesia

## Abstract

**Background:**

Antioxidants located in both the hydrophilic and lipophilic compartments of plasma act as a defence system against reactive oxygen species (ROS). Excessive production of ROS during anaesthesia affects the antioxidant capacity of plasma and may result in oxidative stress. The aim of this study was to evaluate the antioxidant capacity of lipid- (ACL) and water-soluble (ACW) antioxidants in client-owned dogs diagnosed with periodontal disease and early-stage myxomatous mitral valve degeneration (MMVD) and anaesthetised for a dental procedure with propofol and sevoflurane or with propofol only.

**Results:**

Dogs with MMVD were anaesthetised with propofol and sevoflurane (MMVD/PS, *n* = 8) or with propofol only (MMVD/P, *n* = 10). Dogs with no evidence of MMVD (PS, *n* = 12) were anaesthetised with propofol and sevoflurane. Blood samples for determination of ACL and ACW were collected before and 5 min, 60 min and 6 h after induction to anaesthesia. In MMVD/PS dogs, ACL was significantly higher at all sampling times when compared to PS dogs. Compared to basal values, only anaesthesia maintained with propofol significantly increased ACL at 60 min in dogs with MMVD. In MMVD/P dogs, ACW increased after induction to anaesthesia and remained elevated up to 6 h after anaesthesia. Compared to basal values, anaesthesia maintained with sevoflurane significantly increased ACW only at 60 min in both dogs with and without MMVD. The only difference between propofol and propofol/sevoflurane anaesthesia in dogs with MMVD was significantly higher ACW at 60 min after induction to anaesthesia in the propofol group.

**Conclusions:**

Regarding antioxidant capacity, propofol could be a better choice than sevoflurane for anaesthesia of dogs with early-stage MMVD, although further studies are necessary to clarify the advantage of this antioxidant capacity.

## Background

The heart is constantly subjected to ROS formation due to the high rate of aerobic metabolism [1]. Antioxidants located in both the hydrophilic and lipophilic compartments of plasma are actively involved in a defence system against ROS [2]. In dogs with MMVD, a disruption in the balance between ROS formation and antioxidant mechanisms leads to oxidative stress which may contribute to the pathogenesis and progression of the disease [3–6]. During anaesthesia, the metabolism of anaesthetics and changes in tissue oxygenation increase formation of ROS [7, 8] and induce cardiac injury [7, 9] by aggravating oxidative stress [1].

Propofol and sevoflurane are commonly used anaesthetics in dogs. Propofol has a structural feature, the phenolic hydroxyl group, like vitamin E, and is believed to act as a ROS scavenger [10, 11]. Sevoflurane, on the other hand, may promote ROS formation through its metabolism [12] and by influencing mitochondrial function [13].

In order to determine the most appropriate anaesthesia protocol for the dogs with early-stage MMVD, we determined the antioxidant capacity of lipid- and water-soluble antioxidants in dogs with MMVD which were anaesthetised with propofol alone, or in combination with sevoflurane, for a dental procedure. We hypothesised that total intravenous anaesthesia with propofol increases ACL and ACW compared with anaesthesia induced with propofol and maintained with sevoflurane in dogs with MMVD.

## Results

Eighteen dogs diagnosed with MMVD ACVIM class B1 and B2, 7 females (3 neutered, 4 intact) and 11 males (3 neutered, 8 intact), weighing 17.52 ± 8.86 kg and aged 8.65 ± 3.05 years were included in the study. The control group (PS) consisted of 12 dogs with no cardiac disease, 6 females (all neutered) and 6 males (2 neutered, 4 intact), weighing 23.11 ± 9.08 kg and aged 5.88 ± 2.45 years.

Some samples for determination of ACW were excluded from the study due to technical problems with latent fibrin formation and the results are reported only for 7 dogs in the MMVD/PS group, 10 dogs in the PS group and 8 dogs in the MMVD/P group.

There were no differences between the groups in periodontal/dental disease status (*p* = 0.443), antibiotic administration (*p* = 0.443), use of regional nerve blocks (*p* = 0.054), age of dogs (*p* = 0.054) and duration of anaesthesia (*p* = 0.254). Ketamine was administered significantly more frequently (*p* = 0.017) in dogs with MMVD compared to dogs with no cardiac disease.

ACL values were significantly lower in the PS group compared to those in the MMVD/PS group at all sampling times. Compared to basal values, ACL increased significantly 60 min after induction to anaesthesia in the MMVD/P group (Table 1). The ACL value of the propofol formulation used in the study was 3.406 μmol of trolox equivalents per millilitre of propofol solution containing 56.1 μmol of propofol in equal quantity.

**Table 1:** Values of antioxidant capacity of lipid- (ACL) and water-soluble (ACW) antioxidants in dogs diagnosed with myxomatous mitral valve degeneration and anaesthetised with propofol and sevoflurane (MMVD/PS) or propofol (MMVD/P) and in control dogs anaesthetised with propofol and sevoflurane (PS)

Variable	Group	Basal	5 min	60 min	6 h
ACL [nmol/L]	MMVD/PS	133.9 ± 31.3	130.9 ± 29.9	134.3 ± 31.9	131.8 ± 26.2
	PS	87.6 ± 12.2^a^	86.6 ± 13.7^a^	93.0 ± 18.6^a^	84.9 ± 22.2^a^
	MMVD/P	116.4 ± 22.9	118.2 ± 19.9	134.0 ± 23.3*	119.1 ± 24.6
ACW [nmol/L]	MMVD/PS	35.1 ± 8.9	47.1 ± 15.6	52.8 ± 17.2*	42.1 ± 15.5
	PS	29.6 ± 9.6	37.4 ± 4.8	44.3 ± 8.2*	35.44 ± 8.0
	MMVD/P	26.5 ± 9.6	42.5 ± 14.2*	79.2 ± 22.0* ^a^	55.8 ± 30.6*

Data are presented as (mean ± SD). Abbreviations: basal, basal values; 5 min, 5 min after induction to anaesthesia; 60 min, 60 min after induction to anaesthesia; 6 h, 6 h after induction to anaesthesia. ^*^ *p < 0.05* compared to basal values; ^a^ *p < 0.05* compared to MMVD/PS

Compared to basal values, ACW increased significantly 5 min after induction to anaesthesia and remained elevated until 6 h after induction only in the MMVD/P group. At 60 min after induction to anaesthesia, ACW was also significantly increased in the MMVD/PS and PS groups. However, ACW was significantly higher in the MMVD/P group compared to the MMVD/PS group only at 60 min after induction to anaesthesia (Table 1).

## Discussion

In an attempt to elucidate the effect of anaesthetics on oxidative status in dogs with MMVD, this study was set to evaluate the influence of propofol or sevoflurane on plasma water- and lipid-soluble antioxidants in dogs with early-stage MMVD undergoing periodontal treatment under general anaesthesia. The MMVD is the most common acquired cardiovascular disease in dogs with a long preclinical period [14]. Frequently, dogs with an early stage MMVD are diagnosed during a preanaesthetic evaluation, performed for procedures that require general anaesthesia, such as a periodontal treatment. A statistically significant association has been found between periodontal disease and cardiac disease [15].

Metabolism of anaesthetics and changes in tissue oxygenation during anaesthesia promote increased formation of ROS, which affects antioxidant defence system [7, 16, 17]. Propofol and sevoflurane are commonly used anaesthetics in dogs; however, there are only few reports on the effects of these anaesthetics on oxidative stress parameters, including total antioxidant capacity in dogs [18–20] and people [21–23]. So far, ACL and ACW have not been evaluated in dogs during propofol or sevoflurane anaesthesia.

The plasma antioxidant capacity of lipid-soluble antioxidants covers exogenous and endogenous lipophilic antioxidants, including vitamin E, coenzyme Q_10,_ and carotenoids. In the plasma of healthy people, vitamin E represents up to 75% of ACL [24, 25]. A decrease in vitamin E (total tocopherol) during anaesthesia was demonstrated in dogs with MMVD [20], healthy dogs [26] and people [27, 28], as well as a decrease in alpha tocopherol in people [29, 30]. Significantly higher plasma ACL was determined in the MMVD/PS and MMVD/P groups at all sampling times when compared with the PS group in this study. This might mostly be due to increased mobilisation of vitamin E and other lipid soluble antioxidants in response to enhanced ROS production in dogs with cardiac disease [31, 32]. However, we cannot prove that because the individual antioxidants were not measured in our study.

The significant increase of ACL 60 min after induction in the MMVD/P group might be due to the antioxidant properties of propofol. The antioxidant properties of propofol have already been revealed [10, 33–35]. To assess the possible contribution of propofol to ACL, we measured ACL in a sample of the same propofol formulation as we used in the study and established that the propofol formulation has an extremely high ACL, which confirms its antioxidant properties. This is in accordance with studies of antioxidant capacity in human patients during propofol anaesthesia [21, 29, 36]. The lipid soluble component of blood antioxidant activity was evaluated in healthy women, and although propofol had only a small influence on total antioxidant capacity, there was an increase in the antioxidant protection of lipid membranes [36]. Furthermore, propofol accumulates in lipid membranes; thus, plasma may not be the best compartment to evaluate the antioxidant effect of propofol [21].

The antioxidant capacity of water-soluble antioxidants includes hydrophilic antioxidants such as vitamin C, uric acid, glutathione, proteins and low molecular antioxidants [2, 25, 37, 38]. Uric and ascorbic acids are major contributors to the ACW of human plasma, and they are included in the measurement of ACW by using the photochemiluminescence (PCL) method [25, 37–39]. The same method was used in our study. Popov and Lewin [25, 37] reported that ACW determined with the PCL method is age-dependent and animals species-specific. Furthermore, stressors such as exercise, inflammation, or noise resulted in rapid increase of ACW values and ascorbic acid in men [25]. No differences in basal values of ACW were observed between the MMVD/PS and PS groups. These results could be due to homeostasis of water-soluble antioxidant mechanisms was not disrupted in dogs with early-stage MMVD or activation of compensatory mechanisms as enzymatic antioxidant system or increased synthesis of vitamin C. The only difference between the two anaesthesia protocols in dogs with MMVD was at 60 min after induction to anaesthesia, when significantly higher ACW was determined in dogs anaesthetised with propofol. Significantly elevated ACW 60 min after induction to anaesthesia in all groups, and at all sampling times after induction to anaesthesia in the MMVD/P group, may be attributed to the free radical scavenging activity of propofol [10, 34]. Similar results were reported for healthy people anaesthetised with propofol for elective surgery [29]. ACW constitutes mainly of antioxidative capacity of uric acid and antioxidative capacity of ascorbic acid [25, 38]. Uric acid is proposed as a potent free-radical scavenger, the serum concentration of which rises in the settings of acute and chronic oxidative stress [40, 41]. Though less likely, another explanation for a significant increase in ACW in our dogs could be attributed to the increased concentrations of ascorbic acid and/or uric acid as a result of increased oxidative stress during anaesthesia. However, we cannot prove this because the concentration of uric acid or ascorbic acid was not measured in our dogs.

The limitation of this study is that the dogs were client-owned, and thus the environmental and nutritional backgrounds of the dogs differed, which may have influenced the antioxidant parameters [42]. In fact, the antioxidant status may have been affected by different levels of environmental pollution (including, for example, smoking in the household), and although the owners stated that the dogs were not given any vitamins or antioxidants and were mostly fed commercial food, the composition of their food may have differed in antioxidant and vitamin content. Other limitations of the study are the low number of dogs included in the study and the unequal distribution of the number of dogs per group. Because of the low number of dogs per group, post-hoc power analysis was conducted. Statistically nonsignificant results may be the result of insufficient power [43, 44]. Statistical analyses indicated enough power for ACL measurements (power coefficient of 0.843; *n* = 30), while a power coefficient for ACW measurements (0.756; *n* = 25) almost reached the recommended value of 0.80 [43].

## Conclusions

Compared to basal values, ACW was significantly increased 60 min after induction to anaesthesia in both dogs with and without MMVD regardless of the anaesthesia protocol. In dogs with MMVD, ACW was significantly higher in the propofol group. In dogs with MMVD, only anaesthesia with propofol significantly increased ACL. These results suggest that regarding antioxidant capacity, propofol could be a better choice than sevoflurane for anaesthesia, although further studies are necessary to clarify the advantage of this antioxidant capacity in dogs with non-severe MMVD.

## Methods

### Animals

This prospective clinical study was evaluated and approved by the National Ethics Committee and written informed client consent was obtained before the dogs were included in the study. All procedures complied with the relevant Slovenian (Animal Protection Act UL RS, 43/2007) and European regulations. Client-owned dogs receiving no medication 1 month prior to anaesthesia were recruited from the University of Ljubljana Veterinary Faculty subject pool. Their health status was evaluated based on history, physical examination and blood tests including complete blood count with white cell differential count and serum biochemical analyses (glucose, urea, creatinine, sodium, potassium, chloride, calcium, total protein, albumin, alanine aminotransferase, alkaline phosphatase, serum total cholesterol and triglycerides). Cardiovascular disease was confirmed by history, clinical examination, standard electrocardiogram and echocardiography using two-dimensional, M-mode, and colour and spectral Doppler modes (VIVID E9, General Electric Healthcare, Milwaukee, Wisconsin, USA).

All eligible dogs that were presented between June 2016 and January 2017 were included. The final sample size was *n* = 30. The dogs were diagnosed with periodontal disease and scheduled for a dental procedure under general anaesthesia. Eighteen dogs were diagnosed with MMVD class B1 or B2 according to the American College of Veterinary Internal Medicine (ACVIM) classification [45]. They were randomly assigned (by tossing a coin) to anaesthesia with propofol (MMVD/P, *n* = 10) or sevoflurane (MMVD/PS, *n* = 8). The control group consisted of 12 dogs with no evidence of MMVD.

### Study protocol

Dogs were premedicated with morphine 0.3 mg/kg administered subcutaneously (SC) 20 min before induction to anaesthesia. An intravenous catheter was placed into the cephalic vein, and after 5 min of preoxygenation (flow by, 2 L/min), anaesthesia was induced with propofol (3–6 mg/kg) intravenously (IV) and titrated to effect. The dogs were intubated endotracheally within 30 to 60 s, connected to a circle breathing system and allowed to breathe oxygen spontaneously. In dogs with MMVD, anaesthesia was maintained with propofol at 0.3–0.6 mg/kg/min IV (MMVD/P group) or with sevoflurane at an end-tidal sevoflurane concentration of 2 to 3% (MMVD/PS group). Dogs with no evidence of MMVD (PS group) were anaesthetised the same way as the MMVD/PS group. Hartmann’s solution was infused at 5 mL/kg/h IV during anaesthesia. Perioperative analgesia was supported with oral regional nerve blocks with levobupivacaine 1 to 2 mg/kg and/or IV boluses of ketamine 0.5 mg/kg. Ketamin was administered when the heart rate increased by 20% or if signs of nociceptive responses occurred during anaesthesia. Perioperative antibiotic management was carried out with cefazolin 20 mg/kg IV if clinically indicated.

The dogs were placed in dorsal recumbency and warmed with bags filled with warm water. End-tidal sevoflurane concentration, end-tidal CO_2_ tension, respiratory rate, ECG, blood pressure (non-invasively) and rectal temperature were monitored during anaesthesia.

Dogs with the majority of teeth present were included in the study, and a detailed oral examination (probing, charting and full-mouth dental radiographs) was performed prior to periodontal treatment. Dogs were divided into two groups based on their oral/dental disease for statistical analysis: ≤ 25% of the teeth affected with periodontitis and/or dental fractures and > 25% of the teeth affected with periodontitis and/or dental fractures.

During recovery, the dogs were administered Hartmann’s solution at 2 mL/kg/h IV and morphine 0.3 mg/kg SC every 3 h, depending on the invasiveness of the procedure. Carprofen 4 mg/kg IV was administered after the last blood sampling. All dogs were released to home care the same day, with analgesics and antibiotics prescribed as clinically indicated. They were prescribed carprofen 2 mg/kg orally twice daily for 4 to 6 days. In case of severe acute pain, a transdermal fentanyl patch (3 to 4 μg/kg/h) was placed on the skin of the lateral thoracic region at the end of anaesthesia.

### Blood sample collection, processing, and analyses

Venous blood samples were collected from the jugular vein before premedication (basal values) and 5 min (immediate post induction period), 60 min (intraoperative period) and 6 h after induction to anaesthesia (postoperative period).

Blood samples for determination of ACL and ACW concentrations (total volume 2 mL) were collected in lithium heparin containing tubes (Vacuette, Greiner Bio-One, Kremsmunster, Austria). Samples were immediately centrifuged at 1500×g for 15 min at 4 °C and plasma immediately frozen at − 80 °C until analysis.

### Determination of antioxidant capacity of water- and lipid-soluble antioxidants

The method is based on the chemiluminometric detection of photochemically generated superoxide anion radicals from a photosensitizer (luminol), which are partially eliminated from the sample by reaction with antioxidants present in the sample. The remaining radicals react with luminol to produce luminescence, which is measured with a PHOTOCHEM analyser (Analytik Jena, Jena, Germany) using supplied reagent kits. After defrosting and vortexing, 100 μL of plasma sample and 100 μL of methanol were pipetted in a 1.5 mL plastic container with a cap. Samples were then vortexed for 10 min and centrifuged at 25,000 rpm for 10 min at 4 °C. Sample preparation steps were performed in a dark place. Until analysis (usually within 30 min), prepared samples were held in the dark at a temperature below 4 °C. Plasma ACL and ACW were measured in accordance with the manufacturer instructions, using reagent kits (Analytik Jena, Jena, Germany) with the PHOTOCHEM analyser. The results of the ACW measurements are expressed in nmol equivalents of ascorbic acid in mL of the sample, and the results of the ACL measurements in nmol equivalents of trolox (6-hydroxy-2,5,7,8-tetramethylchroman-2-carboxylic acid) in mL of the sample.

To assess whether the propofol formulation used in the study has antioxidative properties, we also measured ACL in a sample of propofol formulation, using the same method as described above.

### Statistical analysis

An a priori sample size calculation was not performed as no comparable data from the literature regarding ACW and ACL were available to enable calculation. Post-hoc sample size calculation indicated that with an effect size of 0.5 and significance level *p = 0.05*, 30 dogs would be enough to achieve more than 80% power of the study. Statistical analysis was supported by the R statistical software program (version 3.2.2) with the nlme package [46]. The Kruskal-Wallis test was used to evaluate the differences in the baseline characteristics of the dogs, age and duration of anaesthesia. Fisher’s exact test was used to evaluate the differences between the MMVD/PS, MMVD/P and PS groups with respect to the baseline characteristics of periodontal/dental disease status and the use of antibiotics, ketamine and nerve blocks. A linear mixed-effect analysis was used to examine the treatment effect and trends over time within groups (MMVD/PS, MMVD/P and PS) and between groups (the MMVD/PS group was compared to the MMVD/P and PS groups). The models included a random intercept for each dog and three fixed effects: time, group and interaction term. In case of significant fixed effects, multiple comparisons were performed using Holm-Bonferroni correction. A value of *p < 0.05* was considered significant.

## Data Availability

The datasets used and/or analysed during the current study are available from the corresponding author on reasonable request.

## Abbreviations

ACL: Antioxidant capacity of lipid-soluble antioxidants
ACW: Antioxidant capacity of water-soluble antioxidants
IV: intravenously
MMVD: myxomatous mitral valve degeneration
MMVD/P: dogs with MMVD and anaesthetised with propofol
MMVD/PS: dogs with MMVD and anaesthetised with propofol and sevoflurane
PS: dogs with no evidence of MMVD and anaesthetised with propofol and sevoflurane
ROS: reactive oxygen species
SC: subcutaneously
